# Head and neck cancer survivors’ preferences for and evaluations of a post-treatment dietary intervention

**DOI:** 10.1186/s12937-019-0479-6

**Published:** 2019-09-10

**Authors:** Sylvia L. Crowder, Katherine G. Douglas, Andrew D. Frugé, William R. Carroll, Sharon A. Spencer, Julie L. Locher, Wendy Demark-Wahnefried, Laura Q. Rogers, Anna E. Arthur

**Affiliations:** 1 Department of Food Science and Human Nutrition, University of Illinois at Urbana-Champaign, 905 S Goodwin Ave, 386A Bevier Hall, Urbana, IL 61801 USA; 2 Department of Nutrition, Dietetics, & Hospitality Management, Auburn University, Auburn, AL USA; 3 Department of Otolaryngology, University of Alabama at Birmingham, Birmingham, AL USA; 4 Department of Radiation Oncology, University of Alabama at Birmingham, Birmingham, AL USA; 5 Department of Medicine, University of Alabama at Birmingham, Birmingham, AL USA; 6 Department of Nutrition Science, University of Alabama at Birmingham, Birmingham, AL USA; 7 Carle Cancer Center, Carle Foundation Hospital, Urbana, IL USA

**Keywords:** Cancer survivors, Diet, Survivorship, Preferences, Barriers

## Abstract

**Background:**

Dietary preferences vary depending on cancer type. The purpose of this study was to report dietary intervention preferences and a study program evaluation from post-treatment head and neck cancer survivors participating in a dietary intervention.

**Methods:**

Between January 2015 and August 2016, 24 head and neck cancer survivors participated in a 12-week randomized clinical dietary intervention trial that promoted weekly consumption of 2.5 cups of cruciferous vegetables and 3.5 cups of green leafy vegetables. At study completion, survivors completed a preferences survey and a study program evaluation to probe interests and improvement aspects for planning future dietary intervention trials. Descriptive statistics (means and frequencies) were generated for multiple choice question responses. Responses to open-ended questions were recorded and grouped based on themes, and verified by quality assurance checks by a second study team member.

**Results:**

Twenty-three survivors completed the preferences and evaluation surveys (response rate 96%). Overall, most participants reported a preference for one-on-one telephone counseling from a registered dietitian nutritionist before beginning treatment. Ninety-six percent of participants ranked the overall study program as “very good” to “excellent,” and all agreed the objectives of the study were clear, the study staff was helpful and easy to contact, and the registered dietitian nutritionist was knowledgeable.

**Conclusions:**

Future research and dietary intervention planning for head and neck cancer survivors should focus on strategies to promote one-on-one telephone or other distance-based counseling combined with face-to-face visits, according to survivor preference.

## Main text

Head and neck cancer (HNC) survivors are at high risk of malnutrition and weight loss after diagnosis due to invasive treatment and resulting symptoms affecting the ability and desire to eat [1]. Additionally, poor quality of life (QOL), reduced functional status, and severe disease- and treatment-related side effects are prevalent in this patient population and often persist long after treatment [1]. The unacceptably low survival rate coupled with the lasting effects of the disease warrant the immediate need for research that focuses on HNC survivorship care [2].

A growing body of evidence suggests nutritional counseling and adequate energy, protein and diet quality are important for improving survival and QOL outcomes in HN and other cancer populations [3–8]. Despite the potential benefits of dietary counseling in HNC, interventions focused on diet quality are limited, and adherence to prescribed diets is poor [9, 10]. Given the high nutritional risk of this population, developing interventions that are appropriate and appealing to HNC survivors are needed in order to optimize participation and adherence. Previous research highlights the importance of incorporating patient preferences to plan interventions in colorectal [11], pancreatic [12], and breast cancer [13] patients. However, dietary intervention preferences in HNC survivors are understudied [8, 14]. As such, the objective of this study was to determine dietary intervention preferences and a study program evaluation as reported by post-cancer treatment HNC survivors who previously participated in a pilot dietary intervention trial [15]. Results can inform future frameworks for successful dietary interventions within this patient population and determine acceptability of research testing dietary interventions.

## Methods

### Design, setting, and participants

Participants were post-treatment HNC survivors enrolled in a 12-week pilot/feasibility dietary intervention trial that took place at the University of Alabama at Birmingham (UAB) Comprehensive Cancer Center from January 2015–August 2016. Participants were randomized to two groups: (1) an attention control group (*n* = 12) that received weekly telephone counseling from a registered dietitian nutritionist (RDN) focusing on general healthy eating for cancer survivors while addressing chronic side effects of treatment that might interfere with the ability or desire to eat; or (2) an experimental group (*n* = 12) who received the same weekly dietary counseling plus additional goals for consuming 2.5 and 3.5 cups of cruciferous (CV) and green leafy vegetables (GLV) per week, respectively. A detailed description of the study population and intervention methods have been described elsewhere [15]. Briefly, stage I–IV oral, pharyngeal, or laryngeal cancer patients who were > 6 months post-treatment and able to consume at least soft foods orally were recruited via the institutional cancer registry. Three-hundred fifty HNC survivors were screened for the study; however, 252 were ineligible or unreachable. Of the 98 eligible participants, 24 consented to participate for a final response rate of 24.4%. Participants completed a survey at the end of the 12-week intervention focused on dietary intervention preferences and an evaluation of the study staff and intervention. All study activities were approved by the UAB and the University of Illinois at Urbana-Champaign (UIUC) Institutional Review Boards (IRB).

### Measures

Patient demographic, behavioral and clinical data were obtained via a health survey. This survey included questions on age, gender, ethnicity, race, education, employment, income, marital status, smoking status, and comorbidities. Clinically measured height and weight were obtained at baseline and follow-up and used to calculate body mass index (BMI). An electronic medical record (EMR) review was conducted to collect clinical data on treatment modality, tumor site, and cancer stage at diagnosis.

### Preferences

After the 12-week dietary intervention, participants completed a 7-item survey regarding their preferences for receiving dietary counseling. Survey questions asked for preferences regarding: 1) from whom they would like to receive dietary counseling (multiple choice; options were physician, nurse, RDN affiliated with a cancer center, RDN at a community center or health club, cancer patient or survivor, and no preference); 2) method of receiving diet counseling (multiple choice; options were face-to-face, telephone, visual communication tool, written material, DVD, interactive workbook, or no preference); 3) size of counseling group (multiple choice; options were one-on-one session or group session); 4) willingness to pay for dietary counseling (multiple choice; options were $0, $1–10, $11–20, $21–30, $31–40, or > $40 per month); 5) willingness to travel for dietary counseling (multiple choice; options were 0 miles, 1–15 miles, 16–30 miles, 31–45 miles, 46–60 miles, or > 60 miles); and 6) ideal timing to start a dietary intervention program (multiple choice; options were before treatment, during treatment, immediately following treatment, 6 months after treatment ends, 1-year after treatment ends, more than 1 year after treatment ends, or other). A final open-ended question asked participants to justify and explain their reasoning behind question 6 (ideal timing to start a dietary intervention program).

### Study evaluations

Participants completed an evaluation of the program at completion of the 12-week intervention. Six questions were rated on a 5-point Likert scale (options ranging from “strongly disagree” - “strongly agree”) including: 1) the objectives of the study and my responsibilities were made clear to me during the initial contact; 2) the study staff was helpful and easy to contact; 3) I would recommend this study to others; 4) the RDN was knowledgeable and helpful; 5) the RDN was respectful and sensitive to how I was feeling at the time; and 6) I would recommend dietary counseling to others. The experimental group had identical questions with the addition of: 1) the RDN counseling sessions were tailored to my needs; and 2) my behavior regarding diet changed during the program.

Participants completed four open-ended survey questions including: 1) what they would change to improve the program; 2) what they would keep about the program; 3) how they would recommend improving the dietary counseling sessions; and 4) how the study could be made more convenient. Participants also responded to one multiple choice question indicating how they would rank the study program overall (options were “poor,” “fair,” “good,” “very good,” and “excellent”). The experimental group had identical questions with the addition of: 1) identifying the part of the program that was most helpful for diet change; and 2) reporting any missed RDN counseling sessions. Additionally, the experimental group was asked whether they participated in a dietary intervention other than the current study in the past 3 months (yes/no).

### Data analysis

Variations in clinical and demographic data and responses to multiple choice survey questions were analyzed using descriptive statistics. For questions with Likert-type response options, “somewhat agree” and “strongly agree” responses were collapsed into one category titled “agree”. Responses of “neutral”, “somewhat disagree”, and “strongly disagree” were collapsed into one category titled “disagree”. Responses to open-ended questions were recorded and grouped based on themes, and verified by quality assurance checks by a second study team member. Inconsistencies in grouping were discussed and resolved.

## Results

### Participant characteristics

Demographic characteristics are presented in Table 1. The mean age of the study participants was 59 years old, and most were Caucasian, males, and college educated. The majority were diagnosed with stage III or IV cancers.

**Table 1:** Characteristics of head and neck cancer survivors participating in a 12-week dietary intervention trial

Characteristic	Total *N* (%)
Age: Mean ± SD [range], years	59 ± 8.2 [44–76]
Gender	
Male	19 (79.2)
Female	5 (20.8)
Race	
Black/ African-American	3 (12.5)
White/ Caucasian	20 (83.3)
Native Hawaiian or Pacific Islander	1 (4.2)
Ethnicity^a^	
Non-Hispanic/Latino	23 (100)
Education	
< High School	9 (37.5)
Some college or more	15 (62.5)
Yearly household income^a^	
(dollars/year)	
> $49,999	11 (47.8)
$50,000 +	12 (52.2)
Tumor Site	
Hypopharynx	2 (8.3)
Larynx	8 (33.4)
Oral Cavity	4 (16.7)
Oropharynx	9 (37.5)
Unknown Primary	1 (4.2)
Cancer Stage	
Stage I	4 (16.6)
Stage II	3 (12.5)
Stage III	7 (29.2)
Stage IV	10 (41.7)
Treatment	
No Radiation	6 (25.0)
Radiation	18 (75.0)
Current marital status	
Married	16 (66.7)
Single (never married)	8 (33.3)
Smoking Status	
Current	2 (8.3)
Past	17 (70.1)
Never	5 (20.8)
Baseline BMI: Mean ± SD [range], kg/m^2	28.1 ± 6.05 [18.5–40.6]
Follow-up BMI: Mean ± SD [range], kg/m^2	28.0 ± 5.87 [19.2–41]

^a^*n* = 23

### Preferences

Table 2 reports preference survey responses among all participants.

**Table 2:** Head and neck cancer survivors’ preferences for dietary counseling assessed post-intervention during a 12-week dietary intervention trial

	*N* (%)	*N* (%)	*N* (%)
	Total (*N* = 23)	Control (*N* = 12)	Experimental (*N* = 11)
*From whom would you most like to receive diet counseling?*			
Registered Dietitian	18 (79)	8 (67)	10 (91)
Physician	1 (4)	1 (8)	0 (0)
Nurse	1 (4)	0 (0)	1 (9)
No Preference	3 (13)	3 (25)	0 (0)
*How would you most like to receive diet counseling?*			
Face-to-face	5 (22)	3 (25)	2 (18)
Telephone	7 (30)	4 (33)	3 (27.3)
Written material (ex: pamphlet, book)	5 (22)	2 (17)	3 (27.3)
Interactive workbook	1 (4)	1 (8)	0 (0)
No preference	5 (22)	2 (17)	3 (27.3)
*How would you prefer to receive dietary counseling sessions?*			
One-on-one sessions	21 (91)	11 (92)	10 (91)
Group sessions	2 (9)	1 (8)	1 (9)
*What is the most you would be willing to pay for dietary counseling? (N = 22)*			
$0/ month	9 (41)	4 (33)	5 (50)
$1–10/month	1 (5)	0 (0)	1 (10)
$11–20/month	4 (18)	3 (25)	1 (10)
$21–30/month	2 (9)	0 (0)	2 (20)
$31–40/month	2 (9)	2 (17)	0 (0)
More than $40/month	4 (18)	3 (25)	1 (10)
*What is the farthest you are willing to travel for dietary counseling?*			
0 miles	1 (4)	1 (8)	0 (0)
1–15 miles	5 (22)	2 (17)	3 (27)
16–30 miles	5 (22)	3 (25)	2 (18)
31–45 miles	0 (0)	0 (0)	0 (0)
46–60 miles	5 (22)	4 (33)	1 (9)
More than 60 miles	7 (30)	2 (17)	5 (46)
*When do you think is the ideal time for head and neck cancer patients to start a dietary intervention/counseling program?*			
Before treatment	11 (48)	5 (42)	6 (55)
During treatment	2 (9)	1 (8)	1 (9)
Immediately following treatment	7 (30)	4 (33)	3 (27)
6 months after treatment	3 (13)	2 (17)	1 (9)

#### Likert-type responses

The majority of participants indicated a preference for dietary counseling from a RDN while the largest proportion of participants preferred to receive telephone-based dietary counseling. Participants preferred counseling to occur before treatment. Nearly all participants indicated a preference for dietary counseling in one-on-one sessions as opposed to group sessions. Nearly half reported they would only be willing to participate in dietary counseling if it were free, and approximately one third indicated they would be willing to travel > 60 miles for counseling.

#### Open-ended responses

The responses for the opened-ended question asking for justification of participants’ choice of ideal timing were categorized into four themes. The majority of participants preferred an intervention before treatment stating reasons such as “overall health and diet” and “the sooner, the better”. Roughly one-third of participants noted the intervention should occur during or immediately following treatment for the “convenience or ease” of the patient as they would already be reporting to clinic and attending follow-up visits frequently. The smallest group of participants preferred an intervention after a post-treatment “adjustment period”.

### Study and intervention (program) evaluation

Table 3 reports program preference responses among all participants.

**Table 3:** Head and neck cancer survivors’ program preferences assessed during a 12-week dietary intervention trial

	*N* (%)	*N* (%)	*N* (%)
	Total (*N* = 23)	Control (*N* = 12)	Experimental (*N* = 11)
*Objectives of study were clear*			
Disagree^a^	0 (0)	0 (0)	0 (0)
Agree^b^	23 (100)	12 (100)	11 (100)
*Study staff was helpful and easy to contact*			
Disgree^a^	0 (0)	0 (0)	0 (0)
Agree^b^	23 (100)	12 (100)	11 (100)
*I would recommend the study to others*			
Disagree^a^	1 (4)	1 (8)	0 (0)
Agree^b^	22 (96)	11 (92)	11 (100)
*Dietitian was knowledgeable and helpful (n = 22)*			
Disagree^a^	0 (0)	0 (0)	0 (0)
Agree^b^	22 (100)	11 (100)	11 (100)
*Dietitian was sensitive and respectful (n = 22)*			
Disagree^a^	0 (0)	0 (0)	0 (0)
Agree^b^	22 (100)	11 (100)	11 (100)
*I would recommend this program to others*			
Disagree^a^	2 (5)	1 (9)	1 (9)
Agree^b^	21 (95)	11 (91)	10 (91)
*Overall, how would you rank this program?*			
Poor	0 (0)	0 (0)	0 (0)
Fair	0 (0)	0 (0)	0 (0)
Good	1 (4)	1 (8)	0 (0)
Very good	9 (39)	4 (33)	5 (45)
Excellent	13 (57)	7 (59)	6 (55)
*Experimental only: Dietitian counseling sessions were tailored to my needs*			
Disagree^a^		–	0 (0)
Agree^b^		–	11 (100)
*Experimental only: My behavior regarding diet changed during the program*			
Disagree^a^		–	2 (18)
Agree^b^		–	9 (82)

^a^Disagree includes strongly disagree, somewhat disagree, or neutral responses

^b^Agree includes somewhat agree or strongly agree

#### Likert-type responses

Overall the study was deemed acceptable to participants. All participants ranked the study program as good – excellent. All participants agreed the objectives of the study were clear, the study staff was helpful and easy to contact, the RDN was knowledgeable and helpful, and the RDN was sensitive and respectful. All but one participants would recommend the study to others and all but two participants would recommend the program to others. All participants in the experimental group agreed the counseling sessions were tailored to their needs. Slightly over 80% of the experimental group participants agreed their behavior regarding diet changed during the program.

#### Open-ended responses

All participants were asked four open-ended questions. When asked what they would change to improve the program, 11 participants provided no response and seven indicated there were no changes needed. Responses from the control group included: “more provider intervention/more contact time”, “a worksheet to e-mail vegetable consumption to the RDN”, and “a location that is closer to home”. One participant in the experimental group suggested “more snack recipes” and another stated they needed more motivation to “follow advice better”.

When asked what should be kept about the program, ten participants provided no response. Responses indicating what to keep about the program included: the entire program (*n* = 7), the colored pamphlets offered to participants (*n* = 3), and the RDN who administered dietary counseling sessions on the phone (*n* = 1). Two participants stated they will retain the skills learned for eating more vegetables and will “eat better”.

When asked how to improve the dietary counseling sessions, ten participants provided no response while ten stated the program did not need improvement. Other responses included clarifications on calories and protein (*n* = 2) and being informed of final study results (*n* = 1).

When asked how the study could be more convenient for participants, 11 provided no response and eight stated the study was already convenient. Four participants indicated it would be more convenient if the program were closer to home.

The experimental group (*n* = 11) was asked three additional open-ended questions. When asked which part of the program was most helpful inducing diet change one participant did not provide a response. Other responses included: “eating more vegetables” (*n* = 8) and the RDN’s availability/phone calls (*n* = 2). When asked if any RDN counseling sessions were missed, three participants provided no response, five stated none were missed, and three stated a counseling session was missed for “travel”, “working late”, and “scheduling conflicts”. No participants reported that they had participated in any other dietary intervention program other than the current study in the past 3 months.

## Discussion

This study reported HNC survivors’ preferences for receiving dietary counseling and their evaluations of a post-treatment dietary intervention aimed at increasing CV and GLV. Notable findings were that the majority of survivors indicated a preference for dietary counseling from a RDN and just under half of survivors indicated a preference for telephone-based counseling and to receive the counseling before treatment.

The preference for telephone-based counseling is a distance-based delivery method and is similar to a study assessing exercise preferences in bladder cancer survivors in which a home-based approach was preferred [16]. Furthermore, a telephone-based distance approach to improve diet quality was utilized in a breast and prostate cancer survivor study in which the intervention arm had a significantly higher diet quality index score as compared to the control arm [17]. Several dietary intervention studies have favored telephone-based methods to provide counseling [17–21] and evidence suggests these distance-based approaches were successful in increasing fruit and vegetable intake in breast [22], colorectal and mixed cancer groups [18]. More so, telephone-based interventions are associated with higher patient adherence in adults with chronic disease [23]. As telemedicine and mHealth applications are becoming more widespread, distance-based approaches such as those offered by telephone- and other distance-based counseling offer a promising method to relieve participant burden, specifically when patients are ill and during periods of increased stress.

The preference for one-on-one dietary counseling in this HNC study is similar to exercise preferences in head and neck [24], pancreatic [12] and breast cancer survivors [25]. This is an important concept to acknowledge in designing future interventions as group counseling may initially appear to be a way of increasing peer support and thus intervention adherence. However, in two previous studies of breast cancer survivors, results indicated that group dietary counseling sessions did not lead to an increase fruit and vegetable intake [26, 27]. In comparison, in a mixed cancer cohort study patients who received individualized dietary counseling during treatment reported better weight maintenance and higher intakes of adequate protein and calories as compared to those in the control arm who did not receive individualized counseling [28].

Just under half of participants responded that they would be willing to receive dietary counseling if it were free. In addition to clinical toxicities, cancer survivors experience financial burden as a consequence of cancer treatment [29]. For example, in the United States, Medicare coverage only includes outpatient medical nutrition therapy provided by a RDN for beneficiaries with diabetes or kidney disease, but health care spending for cancer equals or exceeds these costs [30]. Early preventive and therapeutic nutrition services could prevent malnutrition that is associated with increased risk of treatment toxicities [31], hospital admission and length of stay, and mortality [32] while significantly lowering health care costs [30]. Oncology RDNs are uniquely poised to tackle nutritional issues, including malabsorption, nutrient deficiency, and weight loss/gain that impede oral intake and impact QOL in oncology patients. As the needs of HNC survivors are highly complex, RDNs who have appropriate training in nutrition and dietetics therapeutic counseling are able to effectively manage patients’ nutritional challenges [33]. To meet cancer survivors’ demand for nutrition services and improve nutrition-related outcomes, changes in plan benefit design and coverage policies are needed to encourage access to cost-effective nutrition services across the cancer continuum [30].

In addition to preferences, our evaluation survey results showed the program was acceptable and desirable in HNC survivors as nearly all survivors rated the program as “very good” or “excellent” and would recommend the program and study to others. Furthermore, participants agreed the objectives of the study were clear and the study staff was easy to contact and knowledgeable.

This study adds to the existing body of literature for dietary interventions among HNC survivors [34–36]. Our pilot evaluation data suggests the feasibility and acceptability of testing dietary interventions in this patient population with preferences towards telephone-based intervention methods. However, limitations of the study should be noted. Our sample size is small. However, HNC is a rare cancer type, constituting only 5% of all new cancer diagnoses worldwide, and little information on dietary counseling preferences of this patient population is known [4, 37]. Furthermore, the evaluation methods were purely descriptive and additional qualitative probing of preferences through semi-structured interviews would have increased the robustness of the research findings. The current study utilized open-ended questions. However, some statements assessed more than one result. For instance, “the study staff was helpful and easy to contact” should have been asked as two separate questions. Additionally, this intervention took place 6 months post-treatment while the majority of participants preferred a dietary intervention to take place before treatment. Future interventions should emphasize a pre-treatment intervention in accordance to survivor preferences.

## Conclusion

Results of this study provide information on HNC survivor preferences that should be considered when planning dietary interventions. This is of particular importance as preferences for lifestyle interventions seem to vary depending on cancer type [12, 38, 39]. Study participants indicated a desire for dietary counseling, primarily before treatment. Counseling from a RDN was the preferred delivery source and most preferred a telephone-based delivery mode. The majority of participants indicated they would be willing to receive dietary counseling if it were free, highlighting the importance of insurance reimbursement for oncology nutrition services. RDNs have the ability to successfully manage the various nutritional challenges of HNC patients, thus they are uniquely poised to offer a promising means of managing and improving numerous cancer-related outcomes [33]. Future research should emphasize dietary intervention strategies to promote one-on-one telephone or other distance-based counseling, combined with face-to-face visits, according to survivor preference.

## Data Availability

Not applicable.

## Abbreviations

CV: Cruciferous vegetables
GLV: Green leafy vegetables
HNC: Head and neck cancer
QOL: Quality of life
RDN: Registered dietitian nutritionist

## References

[1] Crowder SL (2018). Nutrition impact symptoms and associated outcomes in post-chemoradiotherapy head and neck cancer survivors: a systematic review. J Cancer Surviv.

[2] Hudson MM, Landier W, Ganz PA (2011). Impact of survivorship-based research on defining clinical care guidelines. Cancer Epidemiol Biomark Prev.

[3] Arthur AE (2014). Diet and proinflammatory cytokine levels in head and neck squamous cell carcinoma. Cancer.

[4] Arthur AE (2013). Pretreatment dietary patterns, weight status, and head and neck squamous cell carcinoma prognosis. Am J Clin Nutr.

[5] Barrera S, Demark-Wahnefried W (2009). Nutrition during and after cancer therapy. Oncology (Williston Park).

[6] Yang Yun-Chin, Lee Meei-Shyuan, Cheng Hsiang-Ling, Chou Hsiao-Yin, Chan Lin-Chien (2018). More Frequent Nutrition Counseling Limits Weight Loss and Improves Energy Intake During Oncology Management: A Longitudinal Inpatient Study in Taiwan. Nutrition and Cancer.

[7] Ravasco P (2005). Dietary counseling improves patient outcomes: a prospective, randomized, controlled trial in colorectal cancer patients undergoing radiotherapy. J Clin Oncol.

[8] van den Berg MG (2010). Comparison of the effect of individual dietary counselling and of standard nutritional care on weight loss in patients with head and neck cancer undergoing radiotherapy. Br J Nutr.

[9] McDonough EM (1996). Relationship between psychological status and compliance in a sample of patients treated for cancer of the head and neck. Head Neck.

[10] Capuano G (2008). Influence of weight loss on outcomes in patients with head and neck cancer undergoing concomitant chemoradiotherapy. Head Neck.

[11] Wright SJ (2017). What are colorectal cancer survivors’ preferences for dietary advice? A best-worst discrete choice experiment. J Cancer Surviv.

[12] Hudnall AM, Arthur JW, Lowery JW (2016). Clinical relevance and mechanisms of antagonism between the BMP and Activin/TGF-beta signaling pathways. J Am Osteopath Assoc.

[13] Phillips SM (2019). Breast cancer survivors’ preferences for mHealth physical activity interventions: findings from a mixed methods study. J Cancer Surviv.

[14] Ravasco P, Monteiro-Grillo I, Camilo ME (2003). Does nutrition influence quality of life in cancer patients undergoing radiotherapy?. Radiother Oncol.

[15] Crowder SL (2019). Feasibility outcomes of a pilot randomized clinical trial to increase cruciferous and green leafy vegetable intake in posttreatment head and neck Cancer survivors. J Acad Nutr Diet.

[16] Karvinen KH (2007). Exercise programming and counseling preferences in bladder cancer survivors: a population-based study. J Cancer Surviv.

[17] Demark-Wahnefried W (2006). Lifestyle intervention development study to improve physical function in older adults with cancer: outcomes from project LEAD. J Clin Oncol.

[18] Hawkes AL (2013). Effects of a telephone-delivered multiple health behavior change intervention (CanChange) on health and behavioral outcomes in survivors of colorectal cancer: a randomized controlled trial. J Clin Oncol.

[19] Morey MC (2009). Effects of home-based diet and exercise on functional outcomes among older, overweight long-term cancer survivors: RENEW: a randomized controlled trial. JAMA.

[20] Kim SH (2011). Randomized pilot test of a simultaneous stage-matched exercise and diet intervention for breast cancer survivors. Oncol Nurs Forum.

[21] Mefferd K (2007). A cognitive behavioral therapy intervention to promote weight loss improves body composition and blood lipid profiles among overweight breast cancer survivors. Breast Cancer Res Treat.

[22] Pierce JP (2007). Influence of a diet very high in vegetables, fruit, and fiber and low in fat on prognosis following treatment for breast cancer: the Women’s healthy eating and living (WHEL) randomized trial. JAMA.

[23] Desroches S (2013). Interventions to enhance adherence to dietary advice for preventing and managing chronic diseases in adults. Cochrane Database Syst Rev.

[24] Rogers LQ (2009). Exercise preferences among patients with head and neck cancer: prevalence and associations with quality of life, symptom severity, depression, and rural residence. Head Neck.

[25] Rogers L (2009). Rural breast cancer survivors: excercise preferences and their determinants. Psycho-Oncology.

[26] Greenlee H (2015). Inverted exclamation markCocinar Para Su Salud!: randomized controlled trial of a culturally based dietary intervention among Hispanic breast Cancer survivors. J Acad Nutr Diet.

[27] Greenlee HA (2013). A pilot randomized controlled trial of a commercial diet and exercise weight loss program in minority breast cancer survivors. Obesity (Silver Spring).

[28] Poulsen GM (2014). Randomized trial of the effects of individual nutritional counseling in cancer patients. Clin Nutr.

[29] Carrera PM, Kantarjian HM, Blinder VS (2018). The financial burden and distress of patients with cancer: understanding and stepping-up action on the financial toxicity of cancer treatment. CA Cancer J Clin.

[30] Dietetics, A.o.N.a. (2016). Patient Centered Nutrition Services Payment Model: An Approach to Incentivizing the Routine Provision of High Quality Nutrition Services.

[31] Santarpia L, Contaldo F, Pasanisi F (2011). Nutritional screening and early treatment of malnutrition in cancer patients. J Cachexia Sarcopenia Muscle.

[32] Tuso P, Beattie S (2015). Nutrition reconciliation and nutrition prophylaxis: toward total health. Perm J.

[33] Gilliard LM (2008). Impact of Interventions by a Registered Dietitian on Nutritional Outcomes in Head and Neck Cancer Patients Undergoing Radiotherapy. Int J Radiat Oncol Biol Phys.

[34] Ravasco P (2005). Impact of nutrition on outcome: a prospective randomized controlled trial in patients with head and neck cancer undergoing radiotherapy. Head Neck.

[35] Capozzi LC (2012). Exercise and nutrition for head and neck cancer patients: a patient oriented, clinic-supported randomized controlled trial. BMC Cancer.

[36] Bossola M (2015). Nutritional interventions in head and neck cancer patients undergoing chemoradiotherapy: a narrative review. Nutrients.

[37] Howlader N (2016). SEER Cancer Statistics Review.

[38] Forbes CC (2015). A comparison of physical activity preferences among breast, prostate, and colorectal Cancer survivors in Nova Scotia, Canada. J Phys Act Health.

[39] Jones LW, Courneya KS (2002). Exercise counseling and programming preferences of cancer survivors. Cancer Pract.

